# Metabolic Biomarkers for Monitoring *in Situ* Anaerobic Hydrocarbon Degradation

**DOI:** 10.1289/ehp.6940

**Published:** 2004-12-08

**Authors:** Lily Y. Young, Craig D. Phelps

**Affiliations:** Biotechnology Center for Agriculture and the Environment, Cook College, Rutgers, The State University of New Jersey, New Brunswick, New Jersey, USA

**Keywords:** anaerobic, biomarker, bioremediation, hydrocarbons, naphthalene, petroleum, toluene

## Abstract

During the past 15 years researchers have made great strides in understanding the metabolism of hydrocarbons by anaerobic bacteria. Organisms capable of utilizing benzene, toluene, ethylbenzene, xylenes, alkanes, and polycyclic aromatic hydrocarbons have been isolated and described. In addition, the mechanisms of degradation for these compounds have been elucidated. This basic research has led to the development of methods for detecting *in situ* biodegradation of petroleum-related pollutants in anoxic groundwater. Knowledge of the metabolic pathways used by anaerobic bacteria to break down hydrocarbons has allowed us to identify unique intermediate compounds that can be used as biomarkers for *in situ* activity. One of these unique intermediates is 2-methylbenzylsuccinate, the product of fumarate addition to *o*-xylene by the enzyme responsible for toluene utilization. We have carried out laboratory studies to show that this compound can be used as a reliable indicator of anaerobic toluene degradation. Field studies confirmed that the biomarker is detectable in field samples and its distribution corresponds to areas where active biodegradation is predicted. For naphthalene, three biomarkers were identified [2-naphthoic acid (2-NA), tetrahydro-2-NA, and hexahydro-2-NA] that can be used in the field to identify areas of active *in situ* degradation.

In this article we illustrate how research in our laboratory and others over the past 10–15 years has advanced our knowledge of hydrocarbons and their degradation under anaerobic conditions. We hope to make the case that this field is mature enough at this point for us to begin applying some of our knowledge to field sites. Our understanding of specific degradation pathways should allow us to develop strategies to intelligently encourage degradation and monitor the progress of *in situ* activity.

Regarding the fate of hydrocarbons in the environment, we know that if contaminants such as polycyclic aromatic hydrocarbons (PAHs) and benzene, toluene, ethylbenzene, and xylenes (BTEXs) are not degraded aerobically, they are likely to be transported into anaerobic regions. This occurs in soils during compaction, in sediments in the marine environment, and in freshwater environments during partition and sedimentation. The question is, what happens to these contaminants in these anaerobic environments? From the results of studies that have been conducted for decades, we understand very well the aerobic fate of these kinds of compounds. Much information is available. We know that the molecules have to be activated by oxygenases (monooxygenases and dioxygenases), and molecular oxygen has to participate in these reactions (Atlas and Bartha 1992). Therefore, there must be different mechanisms for anaerobic organisms. Fortunately, we recently have been able to learn much about these mechanisms. In this article, we review the work that has occurred in the last 10 years, which makes it clear that we know enough to begin applying this information for practical purposes.

## Benzene, Toluene, Ethylbenzene, and Xylenes

Many researchers have demonstrated the anaerobic metabolism of BTEXs (for reviews see Hieder et al. 1999; Phelps and Young 2001). In one such study we conducted a series of screenings of BTEX degradation in different sediments and under different anaerobic conditions (Phelps and Young 1999). The results showed that degradation can be demonstrated for all the BTEX compounds to different degrees under the different anaerobic conditions. All the tested compounds were degraded relatively quickly (loss within 21 days). In addition the profiles of contaminant loss were different between a polluted site (Arthur Kill, New York) and clean site (Tuckerton, New Jersey) and between the estuarine Arthur Kill and freshwater Onondaga Lake (New York). Results such as these emphasize the importance of the prevailing local conditions to BTEX degradation.

**Figure 1:**
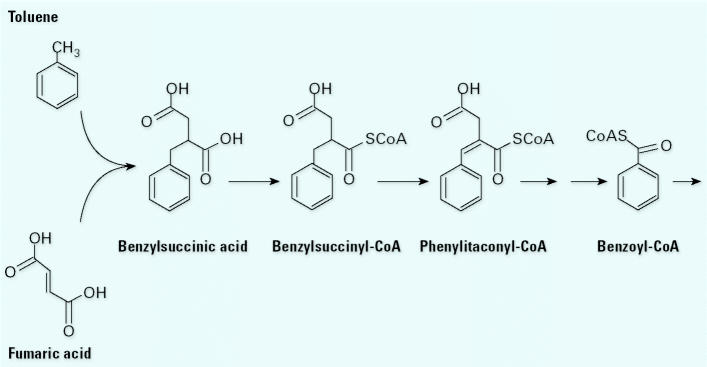
Toluene degradation pathway. The initial reaction of anaerobic toluene degradation involves the addition of fumarate to the methyl group. The resulting benzylsuccinate is activated with CoA, after which it is reduced to phenylitaconyl-CoA. This intermediate then undergoes a series of reactions analogous to β-oxidation to benzozyl-CoA. Figure adapted from Biegert et al. (1996).

Another conclusion from this study is that toluene can be degraded relatively quickly under many reducing conditions (Phelps and Young 1999). This can explain why toluene was the first model compound for anaerobic hydrocarbon degradation and why we know so much about its degradation. In one early study, Evans et al. (1991a, 1991b, 1992a, 1992b) examined toluene degradation under denitrifying conditions. This resulted in isolation of the *Thauera* sp. strain T1 (Evans et al. 1991b), which was one of the first organisms reported that can degrade toluene under anaerobic (denitrifying) conditions. Evans et al. (1991a, 1992a) showed that the toluene could be quantitatively converted to carbon dioxide and cells and that the nitrate was reduced to nitrogen gas. One of their observations that was key in our understanding of BTEX degradation is that when a mass balance for both the nitrogen and the carbon was calculated, the carbon balance did not close completely. The missing carbon was not in the cells, it was not in CO_2_, and it was not left in the substrate. Eventually they determined that it resided in a metabolite, which they then identified as benzylsuccinate, and in variations of benzylsuccinic acid (Evans et al. 1992b). At that time we believed that these were dead-end products and their presence closed the mass balance on the carbon. Since then, Biegert et al. (1996) and other researchers have been able to show that benzylsuccinate is actually a key intermediate in the degradation of toluene. It is formed through a fumarate (4-carbon) addition to the methyl carbon of toluene that activates the molecule. The product of this addition undergoes a series of reactions to produce benzoyl-coenzyme-A (CoA) that then undergoes ring fission and degradation (Figure 1). The discovery of this mechanism was key because the 4-carbon addition turns out to be one of the central reactions in several different pathways for degradation of these and other reduced hydrocarbon compounds.

## Polycyclic Aromatic Hydrocarbons

More recently, researchers have begun to examine the fate of PAHs under anoxic conditions (Coates et al. 1997; Sharak Genthner et al. 1997). In our laboratory Zhang investigated whether PAHs could be degraded under various specific anaerobic conditions using sediment from the Arthur Kill. He was able to show naphthalene, 2-methylnaphthalene, and phenanthrene were degraded under sulfidogenic conditions but not under the other reducing conditions. This initial study demonstrated loss of the parent compounds but not how they are broken down. Zhang and Young (1997) then showed, using ^14^ C-labeled substrate, that from 86 to 92% of that carbon is converted to CO_2_, hence the molecule was being completely mineralized and not merely transformed. They were also able to show that the activity was sulfate dependent. In the presence of SO^=^_4_, naphthalene degradation can occur after 20 hr, whereas in the absence of SO^=^_4_, there is no naphthalene loss. In autoclaved samples there is also no loss. In addition, molybdate, a specific inhibitor of sulfate respiration (Oremland and Capone 1988), prevented the degradation of the hydrocarbon (Zhang and Young 1997). All of this was demonstrated in an enriched consortium, so although it was clear that the activity was sulfate dependent, there was no information about which organism was responsible for the activity.

It is because this consortium of organisms has resisted our attempts to isolate a pure culture capable of metabolizing PAHs that we have had to find alternate ways of working out the degradation mechanisms. Zhang and Young followed an approach of looking for metabolites using gas chromatography-mass spectrometry (GC-MS) analysis. Key to the study of these kinds of consortia was the use of stable isotope–labeled substrates. The label allowed us to conclusively determine that the compounds detected in the culture media were derived from the added substrate. Zhang and Young performed a number of experiments where ^2^H (deuterated) substrate was added to the various cultures. These experiments were used to prove that ^2^H-2-naphthoic acid (2-NA) was produced during metabolism of ^2^H-naphthalene (Zhang and Young 1997).

**Figure 2:**
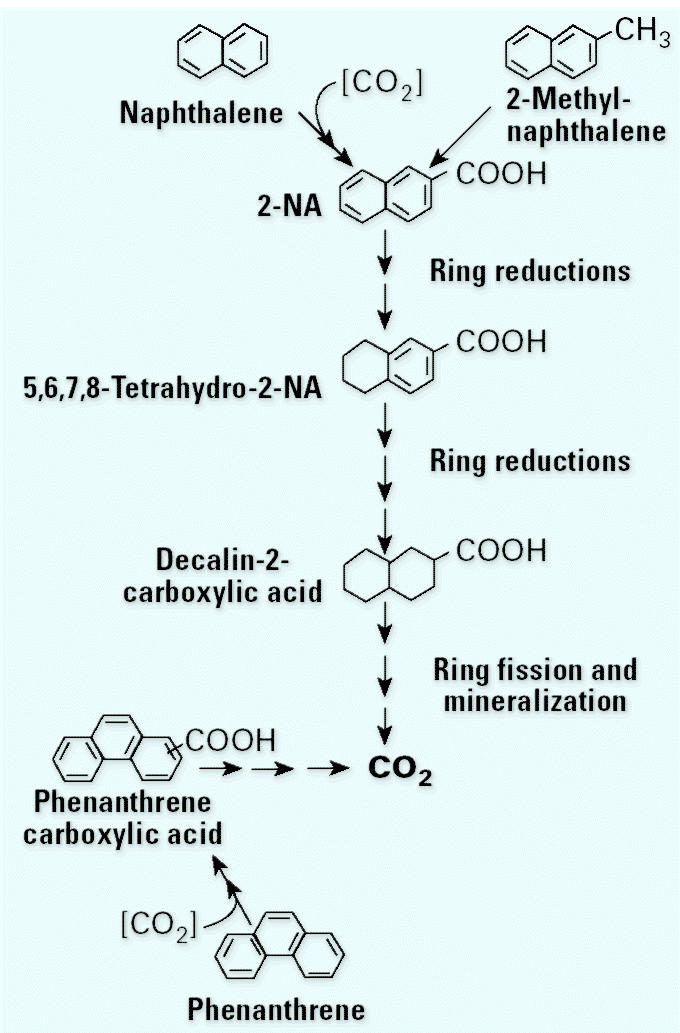
Summary pathways for naphthalene, 2-methylnaphthalene, and phenanthrene. Both naphthalene and 2-methylnaphthalene are initially converted to 2-NA. Naphthalene is transformed by a direct addition of CO_2_, whereas 2-methylnaphtha-lene undergoes a fumarate addition analogous to that seen in toluene. The common intermediate, 2-NA, is further degraded by sequential ring-reduction steps to decahydro-2-NA before ring cleavage takes place. Phenanthrene is also directly carboxylated as the initial step toward mineralization.

Although naphthalene conversion to 2-NA was demonstrated in these experiments using deuterated substrates, the mechanism of carboxylation was still unknown. To determine the source of the carboxyl group, Zhang et al. (2000) added either ^13^C-labeled or unlabeled carbon sources, then looked for the label in the mass spectrum of the resulting 2-NA. When ^13^C-labeled bicarbonate was added, the carboxyl group was labeled ^13^C. This approach was extremely useful in a whole series of experiments. By looking for ^13^C-labeled compounds in cultures amended with ^13^C bicarbonate, Zhang et al. identified a sequence of metabolites during the degradation process. We then put together this sequence of metabolites into a pathway for naphthalene degradation (Figure 2) (Zhang et al. 2000; Zhang and Young 1997). In this pathway the naphthalene is carboxylated to 2-NA followed by a series of reduction steps where hydrogen is added to the unsubstituted ring first and then to the ring with the carboxyl group. This process produces a fully saturated decalin-2-carboxylic acid, which then gets further metabolized to CO_2_. Mineralization was demonstrated with the quantitative recovery of ^14^CO_2_ from cultures fed ^14^C-labeled naphthalene (Zhang and Young 1997).

Using these stable isotope techniques, we were able to work out the fate of 2-methylnaphthalene and phenanthrene in addition to naphthalene (Figure 2) (Sullivan et al. 2001; Zhang and Young 1997). We showed that 2-methylnaphthalene was also oxidized to 2-NA and that the methyl carbon remained on the ring; hence, an external carboxylation did not occur. The resulting carboxylic acid then underwent the same degradation sequence to CO_2_ as observed with naphthalene (Sullivan et al. 2001). Phenanthrene was also carboxylated via the addition of an external bicarbonate molecule. We were not able to confirm the position of this addition because no standards were available (Zhang and Young 1997).

All these experiments were carried out with consortia. Since then, others have confirmed the carboxylation pathway (Annweiler et al. 2002; Meckenstock et al. 2000). In addition, a pure culture that carries out naphthalene degradation in this manner has been isolated (Galushko et al. 1999). Hence, there is increasing evidence that these pathways and mechanisms may be widely distributed in the environment.

## Alkanes

While the PAH degradation mechanisms were being studied, a graduate student in our laboratory was investigating alkane degradation. He was able to isolate an alkane-degrading sulfate reducer that we named strain AK-01 (So and Young 1999b). It is related to strain HXD-3, which is another alkane-degrading sulfate reducer (Rueter et al. 1994). Both strains fall within the same subgroup of delta Proteobacteria. They are both gram-negative, nonmotile sulfate reducers, and they both have very long generation times on the order of several days. However, although they are closely related, they have very different methods of attack on the alkane molecule.

**Figure 3:**
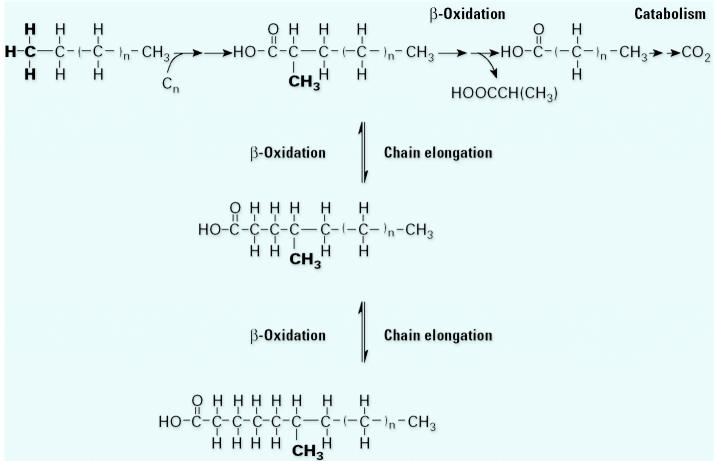
Proposed pathway for anaerobic alkane metabolism by strain AK-01. The terminal methyl group of the alkane substrate is set in bold type to help demonstrate the reaction mechanism. In strain AK-01, alkanes are degraded via an initial attack at the second carbon, resulting in the formation of a carboxylic acid with a subterminal methyl group. The initial attack is presumed to involve fumarate addition. Once the fatty acid is formed, it may be degraded through a number of β-oxidation steps, or it may be incorporated into the cell membrane (So and Young 1999a).

Through a long series of experiments using ^13^C-labeled substrate, So was able to show how hexadecane is anaerobically metabolized by both AK-01 and HXD-3 (So et al. 2003; So and Young 1999a). In AK-01, there is an attack on the subterminal carbon (Figure 3). There is a carbon donor that we could not identify. (Experiments with ^13^C showed that inorganic bicarbonate was not the donor as in the PAHs.) The attack at the C-2 position results in the addition of a carboxyl group and the terminal carbon is displaced to become a methyl group on the subterminal carbon. This was followed by using substrate with a ^13^C label in the C-1 or C-2 position, then analyzing the fatty acids of the cell to determine the position of the label. We showed that the fatty acids in the cells fed hexadecane have a methyl group in the C-2, the C-4, or the C-6 position. The methyl group gets “moved” along the carbon chain by two-carbon additions. Alternatively, the C-1 and C-2 positions can be removed through β-oxidation, and then these two carbons are lost (So and Young 1999a).

**Figure 4:**
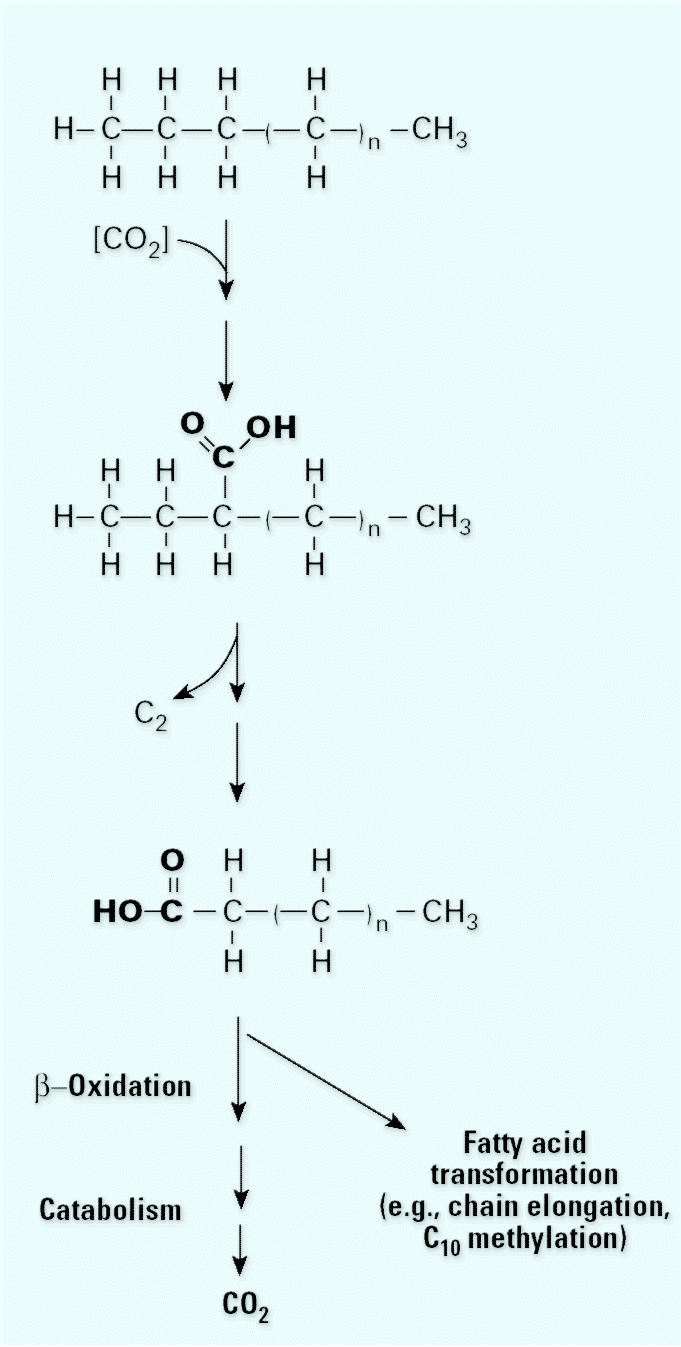
Proposed pathway for the oxidation of alkane to fatty acid by strain HXD-3. The added carboxyl groups are shown in bold type to help demonstrate the reaction mechanism. The mechanism for alkane degradation in strain HXD-3 begins with the addition of CO_2_ to the third carbon of the chain followed by the loss of the two terminal carbons. This results in formation of a fatty acid one carbon shorter than the original alkane. The first intermediate shown in this pathway is hypothetical (So et al. 2003).

In strain HXD-3 a very different mechanism is used. This strain attacks the C-3 carbon of the alkane, then removes the two terminal carbons (Figure 4) (So et al. 2003). Most interestingly, we have shown that the initial attack involves the addition of an external bicarbonate molecule. This was demonstrated using the same stable isotope label techniques as we used to discern the naphthalene degradation mechanism. The resulting fatty acid can then undergo β-oxidation, fatty-acid transformation, or 2-C additions to make larger fatty acids. Thus, we have two different mechanisms of attack on these alkanes by two different but related sulfate reducers.

A recent discovery adds an interesting element to these investigations. It has now been shown that an enriched consortium that grows on dodecane under sulfate-reducing conditions carries out a 4-C/fumarate addition to the C-2 carbon of that alkane (Kropp et al. 2000). On the basis of this finding, it appears that in our hexadecane degrader, AK-01, the carbon donor that we could not identify was actually fumarate, the same mechanism that Evans et al. (1992b) recognized in toluene 10 years ago. In addition, Annweiler et al. (2000) were able to show that the reaction responsible for oxidizing the methyl group of 2-methylnaphthalene to form 2-NA is also a fumarate addition. The mechanism is highly analogous to that which occurs in toluene because of the position of the methyl group on an aromatic ring. What is emerging is a mechanism that appears to be used by a wide range of anaerobic bacteria to activate several very-reduced hydrocarbon compounds that they could not otherwise utilize.

## Summary

There are several methods of attack on hydrocarbon substrates such as toluene, alkanes, and PAHs by anaerobic bacteria. Two of these appear to have very broad specificity to both aliphatic and aromatic hydrocarbons. One method is carboxylation with inorganic carbon. We have demonstrated this mechanism of attack on alkanes, naphthalene, and phenanthrene (So et al. 2003; Zhang and Young 1997). The other method is fumarate addition, seen in toluene, xylenes, methylnaphthalene, and alkane degradation (Annweiler et al. 2000; Beller and Edwards 2000; Beller and Spormann 1997a, 1997b; Evans et al. 1992b; Krieger et al. 1999; So and Young 1999a). These reactions are mediated by anaerobes that are widely different both physiologically and phylogenetically. They have been seen in denitrifyers, sulfate reducers, iron reducers, phototrophs, and methanogenic consortia (Beller and Edwards 2000; Beller et al. 1992, 1996; Rabus and Widdel 1995; Seyfried et al. 1994; Zengler et al. 1999).

All these observations indicate several fundamental mechanisms underpinning anaerobic hydrocarbon degradation. The implications are widespread. An understanding of the basic mechanisms can obviously help us understand processes such as global carbon turnover and biogeochemical processes and provides insight into environmental remediation. The remainder of this article focuses on how we have used this information to develop applications and potential applications for *in situ* biodegradation.

## Biomarkers

For demonstrating *in situ* biodegradation, a National Academy of Sciences (NAS) report in 1993 (NAS 1993) indicated that certain criteria must be met. These criteria include *a*) documenting loss of contaminants from the site, *b*) demonstrating that onsite microbes can transform the contaminant, and *c*) showing that the biodegradation potential is realized in the field. We are interested in using metabolic biomarkers as a means of meeting this third criterion.

An ideal metabolic biomarker should be *a*) formed during active biodegradation of the target compound, *b*) specific to the process being monitored, *c*) normally absent in unimpacted environments; *d*) water soluble for ease of sampling, and *e*) biodegradable. This last criterion may seem counterintuitive from a standpoint of being able to detect the biomarker, but it ensures that the activity being detected is current. If the metabolite is not biodegradable, it is impossible to tell if the activity happened 2 weeks ago or 20 years ago.

## Benzene, Toluene, Ethylbenzene, and Xylene Biomarkers

A convenient aspect of studying *in situ* toluene degradation is that the benzylsuccinate synthase (fumarate addition) pathway is widely distributed. It has been found in denitrifyers, sulfidogens, iron reducers, methanogenic consortia, and even an anoxygenic photosynthetic organism (Beller and Edwards 2000; Beller et al. 1992, 1996; Rabus and Widdel 1995; Seyfried et al. 1994; Zengler et al. 1999). The pathway is well distributed in the environment; therefore, the intermediates can be used as biomarkers under a variety of conditions. Indeed, in 1995 Beller et al. (1995) published an article in which they specifically suggested that the by products of alkylbenzene metabolism could be useful as indicators of *in situ* bioremediation. They showed that toluene and all xylenes have benzylsuccinate-like metabolites and that these can be used as indicators that metabolism is occurring *in situ*. Their demonstration involved making a single bolus injection of BTEXs at the experimental site at Seal Beach, California, and following the loss of the parent compounds and the transient presence of the corresponding fumarate addition products.

We were interested in following this to determine if the same methods could be used to detect ongoing activity at older sites such as abandoned manufactured gas plants (MGPs) common in New Jersey. Rather than search for all the potential toluene and xylene metabolites, we concentrated on measuring the occurrence of 2-methylbenzyl-succinate (2-MBS) because laboratory studies indicated that this isomer accumulated in higher concentrations than the others. Initially, laboratory studies used sediments from Onondaga Lake, which is a freshwater superfund site in Syracuse, New York; Pile’s Creek, a polluted tributary of the Arthur Kill; and Blue Mountain Lake, a nearly pristine lake in the Adirondack Mountains of New York. The results of these experiments showed that in the two polluted sediments where toluene loss was complete, *o*-xylene concentrations declined and 2-MBS was transiently formed. This pattern was consistent with 2-MBS being produced co-metabolically from *o*-xylene during toluene degradation, then being degraded by other organisms in the sediment. No activity was seen in the Blue Mountain Lake sediment, suggesting that the relevant organisms were not present.

## Field Tests

### Site characteristics.

The site chosen for examining 2-MBS as an *in situ* biomarker is located in Glassboro, New Jersey. The contamination present in the groundwater is the result of waste disposal at an abandoned MGP that operated for over 40 years prior to 1951. In the 1980s, all surface and subsurface structures were removed during remediation of the unsaturated soil. The contaminant plume originating from the MGP extends northward from the source through a residential area. Pollutants present include BTEX, PAHs, styrene, phthalates, ethers, and chlorinated solvents.

A great deal of heterogeneity is present in the size of particles in the upper 70 ft of sediment, ranging from gravel to clay. Therefore, the flow of water through this site is expected to be far from uniform. Another result of this heterogeneity is that measurements of the groundwater showed only a general spatial trend toward anoxia, lower redox potential, and increased anaerobic activity in the downgradient direction. It is likely that individual flow paths and areas within the plume are much more reduced and therefore anaerobic. Ion data from individual wells in different parts of the plume did indeed show that, in the area directly downgradient of the contamination, nitrate and sulfate have been depleted and iron has been solubilized, indicating anaerobiosis.

### Sampling.

**Figure 5:**
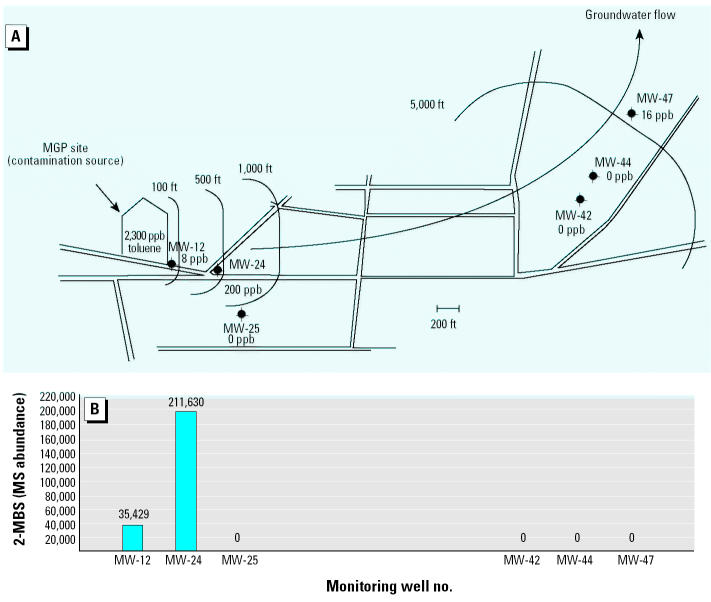
Distribution of 2-MBS in the contaminant plume at the MGP site. (*A)* Diagram of the contaminated site. The MPG, which is the source of contamination, is located at the left; the general direction of ground-water flow is indicated by the arrow. Approximate distances from the MGP site are indicated by the arcs at 100, 500, 1,000, and 5,000 ft. Roads are indicated as parallel lines. Each sample well is shown with the concentration of toluene found at that position at an earlier time. (*B*) graph showing the abundance of 2-MBS at each well. The abundance is expressed as the area of the 351 mass peak from the chromatogram.

Six monitoring wells were chosen for sampling. Two (MW-12 and MW-24) are located within 500 ft of the contaminant source and had measurable amounts of toluene present in the groundwater. Another two of the wells (MW-42 and MW-44) are approximately 4,000 ft downgradient of the source and had no detectable toluene present. One well (MW-47) is located far downgradient from the source and contains some toluene, likely from off-site contamination. The sixth well (MW-25) is located outside the plume area (Figure 5).

Four liters of groundwater were drawn from each sampling site and acidified to a pH < 2 with concentrated hydrochloric acid on site. The samples were stored in precleaned amber glass bottles at 4°C until analysis.

### Extraction and analysis.

A 1-L sample of the acidified groundwater was extracted three times with 200 mL anhydrous diethyl ether. The pooled ether was concentrated under vacuum and passed through anhydrous sodium sulfate to remove any residual water. The remaining solvent was dried under a stream of argon gas. This dry extract was derivatized with bis(trimethylsilyl)trifluoroacetamide (BSTFA; Sigma Chemical Co., St. Louis, MO) according to the manufacturer’s instructions. The derivatized extracts were analyzed by injection into a Hewlett-Packard 5890 series II gas chromatograph coupled to a series 591 mass selective detector (Hewlett-Packard, Wilmington, DE). The column was a J&W Scientific DB-5MS that measured 30 m × 0.25 mm (inner diameter) with a film thickness of 0.25 μm (VWR International, West Chester, PA). 2-MBS was quantified by the area of the 351 ion peak, which represents a derivatized fragment. Benzylsuccinate spiked into groundwater could be measured at concentrations as low as 5 nM. We assume that the recovery of the 2-MBS would be similar.

### Results.

As illustrated in Figure 5, the biomarker 2-MBS was detected in two (MW-12 and MW-24) of the six monitoring wells tested. Both these wells were immediately downgradient from, and closest to, the contaminant source. The abundance of 2-MBS in well MW-24 was approximately 6 times that of well MW-12. This is consistent with the observation that toluene concentrations were also much higher in that well (200 ppb vs. 8 ppb). No 2-MBS was found in wells far downgradient (MW-42, MW-44, and MW-47) or in the well outside the plume (MW-25).

Because toluene is more easily biodegraded than the PAHs that make up the majority of the hydrocarbons present at the MGP site, it is not surprising that it is found only near the source of the plume. We would expect that the toluene and xylenes would be consumed soon after leaving the MGP site. These expectations match very well with the distribution of both toluene and 2-MBS in the plume.

## Naphthalene Biomarkers

### Sampling and analysis.

**Figure 6:**
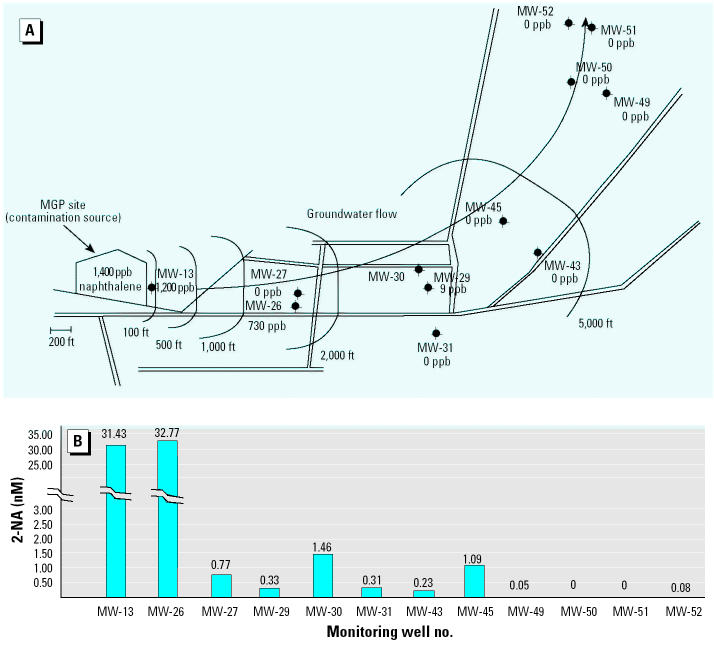
Distribution of 2-NA in the contaminant plume at the MGP site. (*A*) Diagram of the contaminated site. The MGP, which is the source of contamination, is located at the left; the general direction of ground-water flow is indicated by the arrow. Approximate distances from the MGP site are indicated by the arcs at 100, 500, 1,000, 2,000, and 5,000 ft. Roads are indicated as parallel lines. Each of the sample wells is shown with the concentration of naphthalene found at that position at an earlier time. (*B*) Graph showing the abundance of 2-NA at each well.

We examined the same MGP site for evidence of PAH degradation. Samples were taken in the same manner as for the toluene metabolite study. The map in Figure 6 shows the naphthalene concentrations that range from 1,400 ppb at the source to nondetectable far downgradient.

We first showed that it is possible to reliably identify the initial intermediate 2-NA from a groundwater matrix at exceedingly low concentrations using a modification of U.S. EPA method 3510C (U.S. EPA 2000). One liter of acidified water was extracted 3 times with 60 mL methylene chloride. The pooled solvent was concentrated under vacuum and passed through a column packed with sodium sulfate to remove any residual water. After drying under a stream of argon, the samples were dissolved in 100 μL methylene chloride and derivatized with BSTFA following the manufacturer’s instructions. Derivatized samples were analyzed on a Hewlett-Packard 5890 series II gas chromatograph in the same configuration used for toluene biomarkers. The temperature program was capable of separating each of the proposed biomarkers.

Identification of 2-NA was based on GC retention time and comparison of the mass spectral pattern to the spectra of an authentic standard. 2-NA can be quantified with a detection limit as low as 2 μg/L from groundwater.

### Results.

The data show a correlation between naphthalene and 2-NA concentrations (Figure 6). 2-NA is most abundant close to the contamination source and decreases with distance. However, it extends farther than the range of detectable naphthalene, which is expected because it is more water soluble.

Another, much smaller site that we examined is the South Jersey Gas Maintenance Yard (SJGMY) (Phelps et al. 2002). This is the site of an underground gasoline storage tank leak that is much more recent than the contamination at the MGP site. At this site we were able to detect metabolites in addition to 2-NA. The mass spectra of tetrahydro-2-NA (TH-2-NA), hexahydro-2-NA (HH-2-NA), and methylnaphthoic acid (MNA) were identified in samples from this site. High concentrations of all the biomarkers are found in the wells closest to the source of contamination, with much lower concentrations as we go farther downgradient.

To summarize, at the MGP site the highest concentration of 2-NA was found at the wells with high levels of naphthalene contamination, and the concentration declined with distance from the source. Levels of 2-NA were very low far downgradient. At the SJGMY site, which is a newer site, we saw 2-NA, TH-2-NA, HH-2-NA, and MNA detected in wells contaminated with naphthalene. Lower concentrations were seen at nearby wells, but none were found outside the plume (Phelps et al. 2002). The presence of all four metabolites collectively in the naphthalene-impacted wells is evidence that the anaerobic microbial activity is present in these anoxic groundwaters. Although the presence of one compound may not be convincing, we believe that because all four compounds appear and follow the same pattern, there is indeed *in situ* activity.

## Conclusions

The laboratory studies of the past 4 years have provided fundamental information on anaerobic biodegradation pathways for reduced hydrocarbon compounds such as BTEXs, PAHs, and alkanes. These pathways include a series of metabolites unique to the anaerobic degradation of these hydrocarbons. Because they are specific and identifiable with these anaerobic processes, the metabolites can be used to assess the *in situ* biodegradation of these contaminants in anoxic subsurface or perhaps sediment environments. Field samples from several sites show their presence and support the conclusion that microbial degradation of hydrocarbons is actively taking place in these anoxic environments.
